# The Use of the Contamination Index and the LWPI Index to Assess the Quality of Groundwater in the Area of a Municipal Waste Landfill

**DOI:** 10.3390/toxics9030066

**Published:** 2021-03-18

**Authors:** Tomasz Knopek, Dominika Dabrowska

**Affiliations:** Faculty of Natural Sciences, University of Silesia, Bedzinska 60, 41-200 Sosnowiec, Poland; tknopek97@gmail.com

**Keywords:** landfill, groundwater quality, contamination index, landfill water pollution index, Sosnowiec

## Abstract

Environmental degradation caused by the migration of pollutants from landfills is one of the biggest problems for urban areas. Systematic monitoring of groundwater in the vicinity of waste dumps allows for an assessment of the degree of risk to the soil and water environment. In this paper, spatiotemporal variation of groundwater pollution near a municipal landfill in Sosnowiec (southern Poland) was investigated. For this purpose, the monitoring results of five physicochemical indicators from 2014–2019 were used. This study presents an example of the application of the Landfill Water Pollution Index (LWPI) and the *C_d_* Contamination Index. The obtained results indicated that the tested waters were negatively influenced by municipal landfills, especially in the southern part (piezometers P8 and P10). The values of the Contamination Index even reached a value equal of about 1400, while the values of the LWPI index reached 305. Significantly lower values of both indicators were obtained using the results of monitoring studies for other piezometers located upstream from the landfill but belonging to the observation network of a neighboring facility. The indices used permit a determination of the level of groundwater contamination from the described landfill and can be used in similar research areas.

## 1. Introduction

Using a landfill is the most common disposal process for municipal solid wastes [1,2,3,4]. This process involves physical, chemical, and microbial stages during degradation [5] and starts with an aerobic phase [6]. Landfill sites are potential or actual sources of soil and groundwater contamination due to the formation of leachate [7]. In order to reduce the negative impact of landfills on the environment, different methods of preserving the ground are employed. One of the most popular methods of securing landfill sites is sealing the base, slopes, and plateau; indirect and side sealing; cutting off the water flow to the site with simultaneous introduction of a series of water drainage systems; developing systems that speed up decontamination of pollutants; and hardening, stabilization, or vitrification of the ground. Old, closed landfill sites do not usually have seals above the ground or leachate drainage systems. One example of a landfill site that has two sealing zones (a mineral layer and a HDPE film layer) is one in Sosnowiec (southern Poland). In this case, the HDPE foil is additionally secured with a geotextile. The landfill also has a sand filtration layer with a drainage system within it. This means that migration of contaminants into the aquifer and its spreading over long distances takes place there. The scale of the process is evidenced by the control of 684 active municipal landfill sites in 2010, which showed that as many as 49 of them (>7%) did not have any substrate seal [8]. Hence, it is extremely important to conduct reliable monitoring of groundwater quality in the vicinity of even potential sources of pollution [9,10]. There are lots of measures and statistical methods to properly monitor water quality [11,12]. The Polish Regulation of the Minister of Environment of 30 April 2013 (Journal of Laws, No. 523) on landfills, determines the way that groundwater around landfills is monitored (number of observation wells, sampling frequency, minimum range of field, and laboratory tests). The basic range of parameters includes specific electrolytic conductivity (EC), pH, total organic carbon (TOC), Cu^2+^, Zn^2+^, Pb^2+^, Hg^2+^, Cd^2+^, Cr^6+^, and polycyclic aromatic hydrocarbons (PAHs). Unfortunately, in the case of many municipal waste landfills, this range of parameters is insufficient to determine the real risk. This is due to the fact that the metal content in groundwater in the vicinity of municipal landfills is often below the quantification limit. Taking into account such ions as Zn or Cd in the analysis of water quality is often pointless. Similar conclusions can be drawn in the case of PAHs. Leachates from landfills can also involve biologically degradable and nondegradable organic compounds, ammonia nitrogen, or chlorinated salts [13]. Additionally, parameters such as chlorides, sulphates, nitrates, and ammonium are determined.

Predictive models [14], monitoring or geophysical tests [15], an isotopic multitracer approach and laboratory tests [16], spatiotemporal variation analysis of parameters [17], using artificial neural networks [18,19], and various pollution indices [20] are typical methods used for assessing changes in groundwater quality. Due to the fact that metals are typical indicators in groundwater monitoring, the heavy metal pollution index [21] and the metal pollution index [22] are quite popular. In the case of microbiological research, the Horton’s Water Quality Index (WQI) is also an extremely interesting indicator [23].

However, in order to use other, more typical, inorganic indicators of groundwater pollution, the *C_d_* Contamination Index [1,24] and the Landfill Water Pollution Index (LWPI; [25]) were used in this study. These are indicators that take into account high groundwater pollution in the vicinity of municipal waste landfills due to the reference of the measured parameters compared to the natural hydrochemical background or the values of these parameters at the inflow of groundwater to the landfill area.

Backman’s Contamination Index takes into account both spatial and temporal changes in contamination on the basis of total index (*C_d_*), as well as the partial indices calculated for the individual parameters (*C_fi_*), which ultimately make up the cumulative index.

LWPI is an indicator which, in a comprehensible way, shows the degree of landfill impact on groundwater quality. This index allows an assessment of the variability of groundwater monitoring results from a single screening and compares these results with results obtained in a different sampling [26]. This measure takes into account the aforementioned ten parameters which, according to European Union regulation, should be monitored in and around landfills.

The aim of this study was to present changes in the values of the *C_d_* and modified LWPI indices for ten piezometers belonging to the monitoring network of the municipal waste landfill in Sosnowiec (southern Poland) in 2014–2019. In order to calculate the index of groundwater contamination and the landfill water pollution index, five parameters that showed the greatest variation in content and were determined in all piezometers throughout the research period were utilized: EC, SO_4_^2−^, Cl^−^, Na, and Fe. Significant exceeding of the quality standards for these parameters allowed them to be used to assess the quality of groundwater in this area.

## 2. Study Area and Data

**Figure 1:**
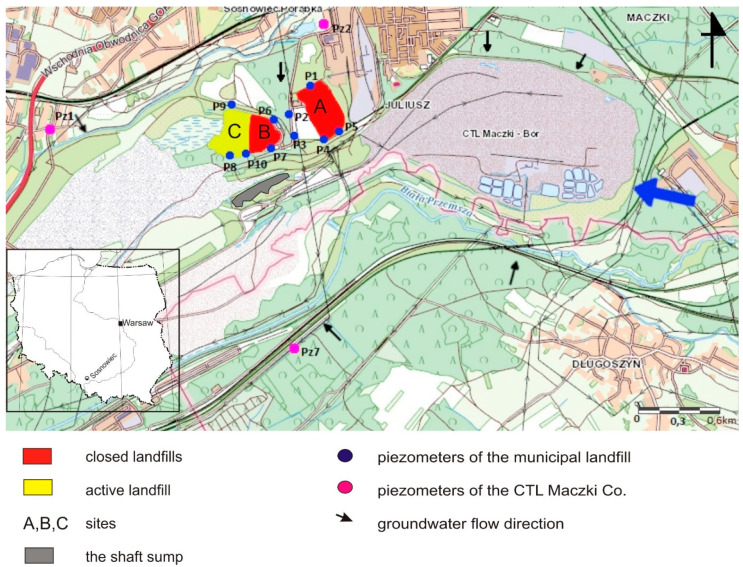
Study area (based on [28]).

The area of the described municipal waste dump is located in the Silesian Province, in the southeastern part of the city of Sosnowiec (southern Poland, Figure 1). The research area, according to the physical and geographic divisions of Poland [27], is located within the mesoregions of the Silesian Upland (341.13; formerly Upper Silesian Industrial District) and Pagóry Jaworznickie (341.14), which are part of the Silesian Upland macroregion (341.1). This area is characterized by a varied topography and has been heavily transformed by local anthropogenic activity. In the area of the landfill, open-cast mining of backfill sand was conducted for the needs of coal mining. The mining excavation of CTL Maczki-Bór, created after the extraction of backfill sand, is liquidated by filling (caving), mainly with waste rock coming from hard coal mines. The area near the municipal waste landfill is drained. The drainage of exploitation slopes and the excavation takes place by a gravity system. The network of ditches and canals changed its course with the progress of mining works and the opening of subsequent layers of the sand deposit, and then with the advancement of reclamation works.

The analyzed area of research is located in the Vistula river basin, within the catchment of the third row—Biała Przemsza.

The municipal landfill in Sosnowiec consists of three sites (A, B, C). Site A has an area of 10.2 ha and is closed. Storage of waste there ceased on 31 March 2006 and it is currently under reclamation. Site B has an area of 4.15 ha and was closed on 10 April 2015. The storage of waste at this site was completed on 31 August 2014. Site C has an area of 4.38 ha and is located in the old excavation. It is technically, technologically, and operationally an extension of site B. Its volume is 580,000 m^3^ [29]. In terms of morphological waste, the dominant fraction is a fine fraction composed mainly of mineral particles (approx. 31%), organic waste (approx. 24%), other mineral waste (approx. 16%), paper and cardboard waste (approx. 14%), and plastics (approx. 10%). The remaining waste is made up of glass, metals, and vegetable waste. Operation started there on 3 June 2013. Additionally, the landfill has a storage facility for construction waste containing asbestos.

In terms of hydrography, the area is located in the catchment of the Biała Przemsza River, belonging to the Vistula basin. The water quality of surface watercourses is generally severely degraded by discharges of untreated industrial wastewater. The Biała Przemsza River is supplied by its tributaries and from mine water discharges from zinc and lead ore mines, open-cast sand mines, and subordinately liquidated coal mines [29]. The river bed does not have a natural character in this area, but has been concreted.

**Figure 2:**
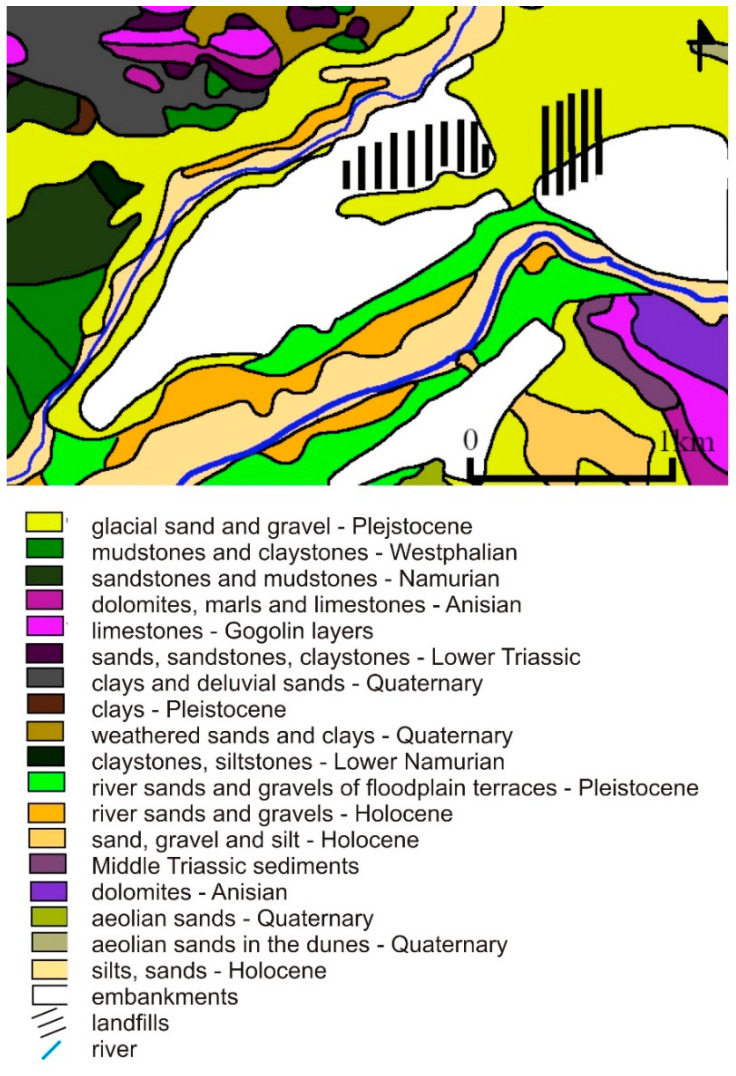
Geological map of the study area [30].

The geological profile of the area includes Upper Carboniferous, Triassic, and Quaternary deposits (Figure 2). The oldest documented Carboniferous formations are Grodziec Beds, represented by alternating medium and fine-grained sandstones, and silty mud formations with numerous thin coal seams. Above, there are Saddle Beds made mainly of sandstone and clay rocks with a 510 carbon seam, with a thickness ranging from a few to approx. 30.0 m. The Ruda Beds are mostly thick-bedded fine, medium, and coarse sandstones, separated mainly by mudstones, with numerous coal seams with a total thickness of approx. 60.0–100.0 m. The maximum thickness of the mud series in this area is up to approx. 540.0 m. It is represented by clay and mud rocks, with numerous coal seams [30].

The Triassic formations are represented in this area by sediments of Buntsandstein, Roethian, and Muschelkalk. The lower Buntsandstein is built by multigrained sandstones with a predominance of coarse-grained sandstones, and sometimes conglomerate ones. The thickness of the discussed deposits is variable and reaches a maximum of 20.0–25.0 m.

The Roethian formations are formed as a complex of gray-green marls, limestones, and pelitic dolomites with thin inserts of marl and clay. The thickness of these formations does not exceed 30.0 m.

The Muschelkalk is represented by plate and marly limestones with local marl inserts. These sediments are often cracked. Crushed dolomites of middle Muschelkalk occur in the form of dolomitic limestones and dolomites. The total thickness of the Muschelkalk is 90.0–95.0 m.

**Figure 3:**
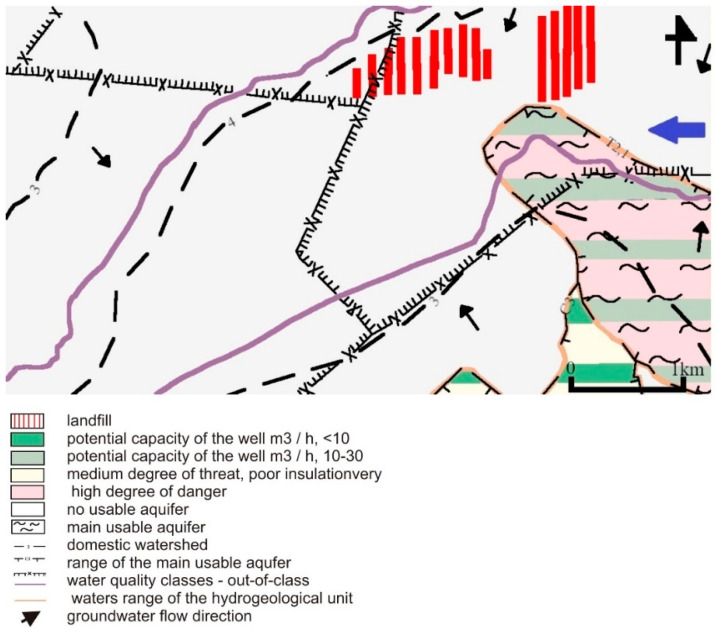
Hydrogeological map of the study area [34].

Sands of various granulation dominate in the profile of the Quaternary formations. Gravel, clay, and clay deposits are secondary in the form of interlayers and lenses, mainly in the lower part of the profile. The Quaternary thickness varies within quite large limits, from several to several dozen meters, depending on the form of the Subquaternary surface [31]. According to the hydrogeological regionalization proposal of Poland according to groundwater bodies [32], the research area is located in the Vistula province, in the Middle Vistula region, in the western part of the Central Vistula upland subregion. In this research area, in the hydrogeological profile, there are Quaternary, Triassic, and Carboniferous aquifers (Figure 3) [33].

The Quaternary aquifer is mainly composed of multigrained sands with gravel inserts and of gravel with sand. There is one main aquifer divided in places into two or three aquifers. The supply of the Quaternary formations takes place mainly through the infiltration of precipitation. Under the conditions of the undisturbed regime, Pleistocene aquifers were mainly supplied by direct infiltration of precipitation and drained exclusively by the Biała Przemsza River. Currently, in the conditions of the groundwater regime disturbed by the activity of mainly opencast mining, a change in the conditions of rainwater infiltration and a change in water circulation routes in the Quaternary formations has been observed. The supply of the Quaternary formations takes place mainly through the infiltration of atmospheric precipitation. The Quaternary formations have hydraulic conductivity in the order of 10^−4^ m/s. The water table is unconfined. The analysis of the contour map for the region of the industrial area shows that the main drainage center of the excavation is the shaft sump. In the area of the drainage basin, the water table in the Quaternary formations lies at the ordinate of approx. +221.0 m. The ordinates of the groundwater table within the excavation are, on average, approx. +225.0–+235.0 m. The minimum thickness of the aquifer is approx. 1.0–3.5 m. The value of depression in the center of the mining excavation is approx. 27.0 m. The average hydraulic drop is 0.025 [31].

The low-thickness Triassic aquifers in this area are poorly explored and have no practical impact on the watering of the nearby excavation. Locally, they can form a common Quaternary–Triassic level [34]. As in the case of the Quaternary layer, the recharging of the Triassic horizons is mainly due to the direct infiltration of precipitation on outcrops or indirectly from the Pleistocene aquifers.

The occurrence of waters in the Carboniferous formations is associated with shoals of sandstones, most often fine and medium-grained, lying within the series of clay and mud layers of Orzeskie, Załęskie, Ruda, and Saddles Beds. The sandstones show a significant diversification of aquifer depending on the depth of deposition and the degree of their tectonic transformation. The values of hydraulic conductivity are in the range of 1.15 × 10^−3^–6.87 × 10^−8^. Recharge is carried out by infiltration through the permeable Quaternary formations or directly on outcrops of the permeable formations of the productive Carboniferous ones [31].

The monitoring network of the Quaternary aquifer in the area of the landfill consists of 10 active piezometers with the symbols: P1, P2, P3, P4, P5, P6, P7, P8, P9, and P10 (Figure 1). The P9 piezometer is located upstream of groundwater from the north. All piezometers are 35–36.2 m deep. The piezometers were drilled beyond the reach of the workings. Their profiles contain the entire range of sands that have been mined, i.e., from fine and medium to coarse sands. The piezometers have a filter section about 2.5 m long, which sits from about 1 m to about 13 m below the water’s surface. The filter does not cover the entire aquifer. Two of the piezometers, i.e., P9 and P10, have a 5 m filter. The filter is a perforated pipe wrapped in wire and mesh.

The database includes the results of chemical analyses for 2014–2019 in the range of redox potential (Eh), pH, EC, Na, Mg, K, Ca, Mn, Fe, TOC, HCO_3_, Cl, SO_4_, and NH_4_. The exception is the database for the P9 piezometer, which only includes data from 2017–2019. Piezometer tests are performed quarterly—in January, April, July, and October. No seasonality appeared from the available data.

All determinations of physicochemical parameters were performed in a certified laboratory. The sampling techniques were developed on the basis of the PN-ISO 5667–11-2004 standard, i.e., quality assurance and quality control guidelines for sampling and handling environmental waters. In order to keep the samples representative, special attention was paid to pumping out three volumes of stagnant water in the piezometer. In order to ensure the quality of the measurement, the samplers constantly monitored changes in EC, pH, and temperature. Sampling was performed using the MP1 Eijkelkamp pump.

Hydrochemically [35,36], the groundwater in these piezometers ranges from three-ion (P10) to even six-ion (all other piezometers). The water in the P10 piezometer can be classified as SO_4_-Ca-Mg, and in the remaining piezometers, for the most part, water of the HCO_3_-SO_4_-Cl-Mg-Ca-Na type can be observed. The mean values of the measured parameters for the analyzed test period are summarized in Table 1.

Five of the above-mentioned parameters (EC, SO_4_^2−^, Cl^−^, Na, and Fe) were used for further research. This range is due to the fact that the analyzed area is characterized by pollution caused by these components. In the case of EC, this parameter was chosen as a typical measure of water mineralization.

To calculate the indicators, the average values of the parameters for the P9 piezometer were taken into account as the background values, as were the data from the Pz-2 bis and Pz-7 piezometers, located in the direction of groundwater flow towards the area of the analyzed landfill but belonging to a separate observation network (CTL Maczki-Bór mine stock company).

## 3. Methods

To evaluate the variability of temporal and spatial changes in the chemical composition of groundwater in the vicinity of the municipal waste landfill in Sosnowiec, the *C_d_* Contamination Index and the Landfill Water Pollution Index were used.

The values of the indices were calculated on the basis of five parameters: EC, SO_4_^2−^, Cl^−^, Na, and Fe. The selection of these parameters was made based on the analysis of monitoring data and the largest number of exceedances of the parameter values compared to the standards for quality class IV (Regulation of the Minister of Maritime Economy and Inland Navigation of 11 October 2019 on the criteria and method of assessing the condition of groundwater bodies, Journal of Laws 2019, item 2148).

The first index was first introduced by Backman et al. (1998). The index is a measure of the pollution reflecting the level of pollutants in groundwater in relation to the natural hydrochemical background [1,24].

The contamination index (*C_d_*) for groundwater in the area of the described parameters was calculated based on the following formula [37]:

(1) $$C_{d}=\sum_{i=1}^{n}C_{fi}$$

where:

(2) $$C_{fi}=\frac{C_{Ai}}{C_{Ni}}-1$$

in which *C_fi_* is the contamination index for the *i*-th parameter, *C_Ai_* is the analyzed value for the *i*-th parameter, and *C_Ni_* is the upper range of the values of the natural hydrochemical background.

The second index takes into account the relationship between the values of individual parameters measured at the observation point and the values for points beyond the influence of the object (upstream groundwater). The LWPI index was calculated using the formula:

(3) $$S_{i}=\frac{C_{p}}{C_{b}}$$

where *C_p_* is the concentration of the *i*-th parameter in each of the polluted groundwater samples and *C_b_* is the concentration of the *i*-th parameter in the inflow groundwater sample (background concentration).

Ultimately, the formula for calculating the ratio looks like this [26]:

(4) $$\mathrm{LWPI}=\frac{\sum_{i=1}^{n}S_{i}w_{i}}{w_{i}}$$

where *w_i_* is the weight of the *i*-th pollutant variable and *n* is the number of groundwater pollutants.

The weight values were calculated for parameters on the basis of [26,38]. The selection of weights to calculate the LWPI was made on the basis of the data contained in the publications. They indicate that the conductivity value should not have such an impact as, for example, the value of other chemical parameters. The weights for the remaining parameters were adopted based on the hydrogeological knowledge of groundwater in the vicinity of pollution dumps, while also taking into account which of these parameters are macronutrients in the waters. Detailed data are presented in Table 2.

The value of the contamination index increases along with an increase in the concentration of individual parameters. In the case of heavily transformed areas, the value of the index exceeds 3 [39]. When LWPI value is ≤1, it describes water under no landfill impact, the second situation (1 < LWPI ≤ 2) describes moderately polluted water due to minor landfill impact, and the last one (2 < LWPI ≤ 5) describes poor water with a highly visible landfill impact. The worst situation is in the case when LWPI > 5, which is characteristic for strongly polluted water [26].

The obtained values of both indicators for the observation network of the described municipal waste landfill were used to create a point map of the values of these indicators in the analyzed area for years 2014–2019. Maps with marked values of both indicators at vantage points allowed us to determine the zones of more polluted waters. The choice of the point-based presentation of the indicator values for such a hydrogeologically complex area allowed for a more reliable determination of the pollution than in the case of interpolation methods [39].

## 4. Results

The calculations of the contamination index (*C_d_*) and LWPI index made for EC, SO_4_^2−^, Cl^−^, Na, and Fe for the groundwater monitoring results from the region of the landfill for 2014–2019 confirmed the negative impact of this landfill on groundwater. The values of *C_fi_* for these individual parameters varied from −1.0 to 2719 (Appendix A). The highest values of partial pollution indicators and the total indicator were obtained using the Pz7 piezometer to calculate the background value. The most favorable values of these indicators were obtained with the Pz2 piezometer. Both of these piezometers belong to the CTL Maczki observation network. Using the P9 piezometer to calculate the hydrochemical background belonging to the observation network of the municipal landfill, we obtained results that indicated pollution, but did not significantly increase the value of the index.

Two additional variants of the hydrochemical background were considered to determine how differentiated the assessment of groundwater pollution degree for the same region can be.

**Figure 4:**
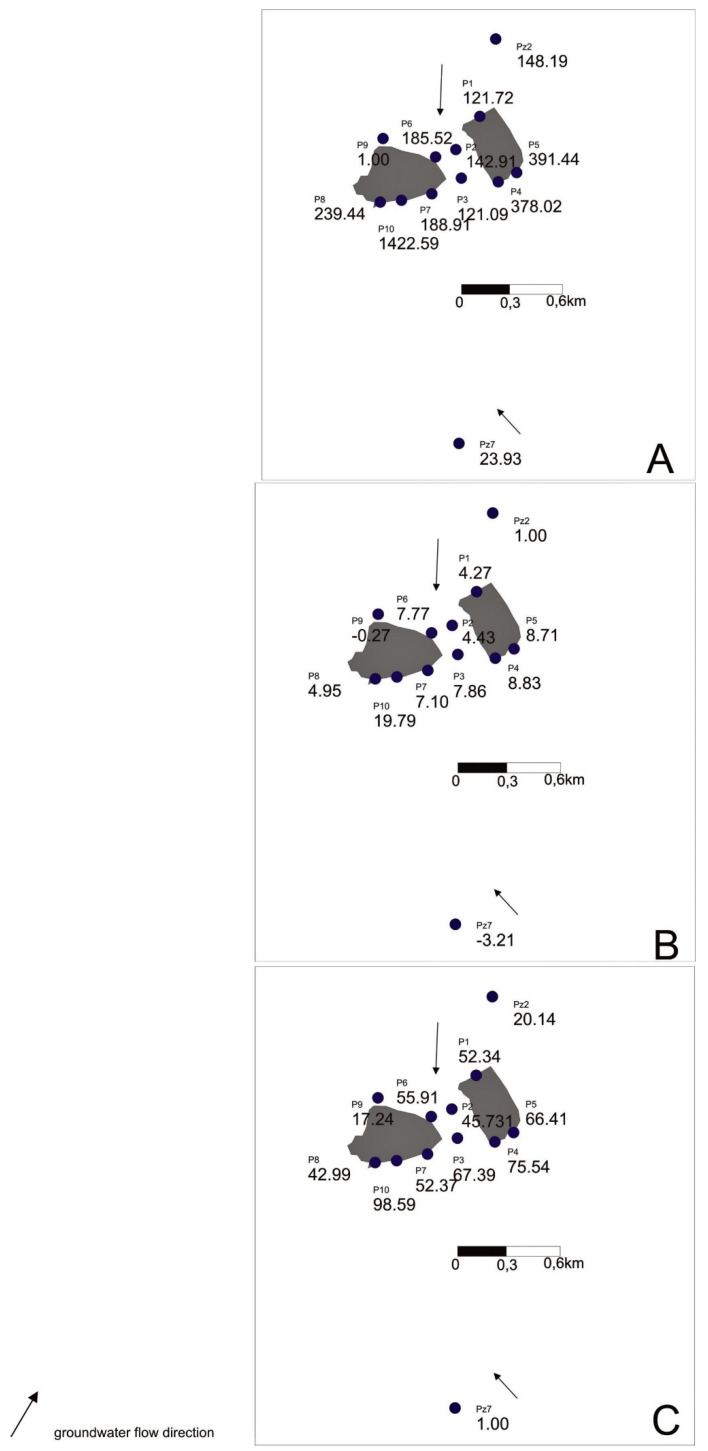
Values of the Contamination index using the results from different piezometers as hydrochemical background: (**A**) P9, (**B**) Pz2, (**C**) Pz7.

The indicators showed the lowest variability for electrolytic conductivity and the highest for iron ion. Groundwater in the landfill area showed the highest pollution in terms of iron and sodium content. The values of partial contamination factors for iron were in the range 279–2719. The lowest value was obtained for the P7 piezometer and the highest for the P10 piezometer (Figure 4). The indices for sodium ranged from about 45 (P7 piezometer) to about 128 (P3 piezometer).

In the case of the total values of pollution indicators, the highest values were obtained using the results from the P9 piezometer as a background. The highest value was obtained for the P10 piezometer—around 1422 (Table 3).

The lowest values were obtained when using the results from the Pz2 piezometer as background. The lowest value of the index was obtained for the P1 piezometer—about 4. Higher values of pollution indicators were observed in the southern part of the landfill.

It is worth noting that the assessment of groundwater pollution in the vicinity of the described municipal waste landfill site with the use of monitoring results from three different piezometers located in groundwater upstream of the landfill gave very diverse results and definitely influenced the interpretation of the results.

The values of the contamination index using the results from the P9 piezometer as background were on average 33 times higher than the results when the Pz2 piezometer was used as the background. For example, the index values for the P3 piezometer in the first variant were only 15 times higher, but for the P10 piezometer this value was over 70 times higher. Such different values for individual variants also affected the appearance of the values of this index (Figure 4A–C).

The LWPI values for individual piezometers also indicate contamination of groundwater in the vicinity of the described landfill. The lowest value of the LWPI in the first variant was recorded for the P3 piezometer—about 8—and the highest value was for the P10 piezometer—about 306 (Table 4). Taking the results from the Pz2 piezometer as the hydrochemical background, we obtained the highest value of this indicator, equal to approximately 5.5. The values of this index when using the first variant were about 20 times higher than in the second variant. The values of the LWPI in the first variant were from about three times higher in the P3 piezometer and up to 56 times higher in the P10 piezometer than in the second variant. The very high values of the LWPI were mainly due to the high concentration of iron ions, for which the weight was 3.

Using the first variant of calculations, the lowest index was calculated for the P3 piezometer; in the second variant, it was the P1 piezometer; and in the third variant, it was the P8 piezometer (omitting the values for the P9 piezometer). The greatest diversification of the indicator values occurred in the first variant. The highest value of the index in this variant was 35 times greater than the smallest. In the case of the second and third variants, these ratios were 3 and 2.4, respectively.

It is worth noting that the EC value, which is the indicator of dissolved inorganic ions in groundwater, does not significantly affect the value of the indicator due to having a weight equal to 1. The EC value for the tested piezometers reached an average of 3000 µS/cm. The highest values were observed in the P3 piezometer, which would suggest that this piezometer captures the most polluted water. However, the highest values of the index were calculated for the P10 piezometer.

The indicators of water transformation in the landfill area mainly include sulphates and an increase in chloride content (factors related mainly to anthropogenic impacts), as well as in iron—this factor may also have a geogenic origin in this environment. Due to the origin of the content of sulphates and chlorides, they were assigned a weight of 4. High values of the index were also caused by a high concentration of sodium ions (even with the assigned weight equal to 2).

**Figure 5:**
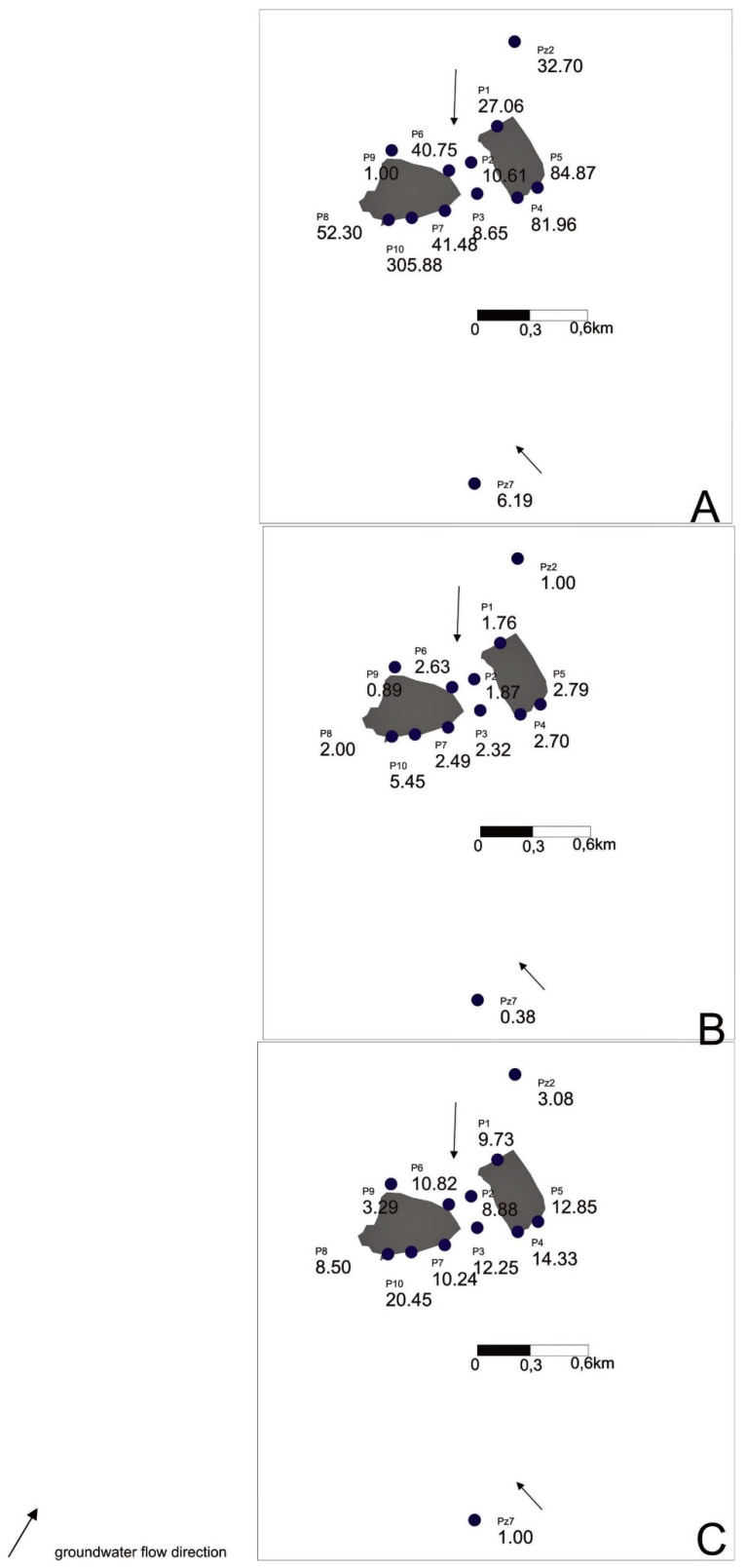
Values of the LWPI index using the results from different piezometers as hydrochemical background: (**A**) P9, (**B**) Pz2, (**C**) Pz7.

Also, the obtained values of the index for various variants of the hydrochemical background may make an unambiguous interpretation of the distribution of pollution difficult (Figure 5A–C).

Both indicators can be the basis for further research on the degree of groundwater pollution in the analyzed area as well as for the analysis of the risk for groundwater. Illustrative maps of pollution can also be part of reports on the quality of groundwater in the area of pollution sources.

## 5. Conclusions

The obtained results revealed that the quality of the groundwater in the region of the analyzed landfill has been impacted by this pollution source. The values of most parameters were higher in downstream groundwater than in the case of groundwater upstream of the landfill.

The calculated values of the Contamination Index and the LWPI index indicated the most negative impact of the landfill in the southern part of the study area (piezometers: P8, P10). The use of other piezometers to be used as reference values for the hydrochemical background resulted in a completely different picture of the degree of groundwater pollution in the described area. The highest values of the indicators were calculated using the monitoring results from the P9 piezometer as a hydrochemical background, belonging to the observation network of the municipal landfill. Another interpretation of the degree of water pollution in this area can be made using the data from piezometers located upstream of waters from the southeast (Pz7 piezometer) and north (Pz2bis piezometer).

The research results presented in this paper indicate that both indicators can be used to assess the quality of water in the vicinity of pollution sources, but the following should be carefully analyzed: the choice of piezometer to determine the hydrochemical background, the choice of individual indicators for the calculation of the summary indicator, and, in the case of the LWPI indicator, the weights assigned to individual parameters.

The values of the *C_d_* index exceeding 1400 and the LWPI values exceeding 300 testify to the high pollution of groundwater in this region.

The pollution index *C_d_* turned out to be more sensitive to changes in the background value (selection of a different piezometer) for this particular landfill. Making value distribution maps for both indices is extremely helpful in interpreting the results from monitoring.

Selecting only heavy metals for the interpretation of contamination in a given region can lead to the water quality being misjudged. When selecting individual parameters, one should be guided by the hydrogeological knowledge of a given area, as well as taking into account various factors that may affect the increased concentration of individual parameters. The value of the LWPI can be more manipulated by assigning different weights to the individual parameters, and hence the interpretation of the degree of contamination may not be objective. Due to the use of higher weights for individual ions, a much greater variation in index values is obtained.

In further work, the possibility of combining both indicators and performing a similar interpretation for the assessment of water quality in this area should be considered.

## Figures and Tables

**Table 1:** Mean values of measured parameters in the monitoring network.

Parameter	Unit	P1	P2	P3	P4	P5	P6	P7	P8	P9	P10
Water table level	m b.g.l	23.868	28.512	29.876	30.791	28.184	19.126	19.104	19.099	23.628	19.358
Eh	mV	154.608	160.638	189.023	159.646	160.172	169.738	164.428	159.472	297.167	140.352
pH	-	9.624	10.654	10.792	10.415	6.944	6.928	6.872	7.024	7.092	6.860
EC	µS/cm	1784.2	1765	3482.923	2377.654	2247.440	2319.200	2170.480	1847.120	1860.333	2692.520
Na	mg/L	252.76	202.154	327.077	284.038	250.040	213.760	194.560	155.796	178.917	179.992
Mg	mg/L	36.648	59.458	138.715	48.969	43.240	105.104	62.108	31.410	41.917	93.424
K	mg/L	14.6	20.512	153.254	16.845	9.165	48.540	19.032	12.968	61.083	13.074
Ca	mg/L	62.4	94.515	101.712	187.862	204.920	151.596	200.960	245.680	193.833	316.120
Mn	mg/L	0.58436	4.775	4.521	11.748	1.141	1.875	27.202	2.236	1.817	4.884
Fe	mg/L	7.832	12.762	9.478	22.764	20.336	10.141	9.590	13.034	0.055	70.122
TOC	mg/L	3.14	7.785	46.181	17.373	3.504	7.144	4.782	3.796	4.945	4.076
HCO_3_	mg/L	344	360.885	1106.923	454.692	433.760	521.240	498.640	693.040	480.750	567.200
Cl	mg/L	319.16	294.577	417.192	483.308	387.320	355.120	328.800	213.872	249.667	321.120
SO_4_	mg/L	190.236	253.654	239.731	214.538	350.720	426.400	374.600	292.280	351.667	829.920
NH_4_	mg/L	0.2718	5.153	81.677	7.239	0.818	5.117	4.842	3.281	0.202	3.966

**Table 2:** Weights of parameters required to calculate Landfill Water Pollution Index (LWPI)**.**

Parameter	Weight (*w_i_*)
EC	1
Na	2
Fe	3
Cl	4
SO_4_	4
Sum of weights	14

**Table 3:** Mean value of the contamination index with three different piezometers taken as the hydrochemical background.

Piezometer	*C_d_* with P9	*C_d_* with Pz2	*C_d_* with Pz7
P1	121.72	4.27	52.34
P2	142.91	4.43	45.73
P3	121.09	7.86	67.39
P4	378.02	8.83	75.54
P5	391.44	8.71	66.41
P6	185.52	7.77	55.91
P7	188.91	7.10	52.37
P8	239.44	4.95	42.99
P9	1.00	−0.27	17.24
P10	1422.59	19.79	98.59
Pz2	148.19	1.00	20.14
Pz7	23.93	−3.21	1.00

**Table 4:** Mean value of the LWPI index with three different piezometers taken as the hydrochemical background.

Piezometer	LWPI with P9	LWPI with Pz2	LWPI with Pz7
P1	27.06	1.76	9.73
P2	10.61	1.87	8.88
P3	8.65	2.32	12.25
P4	81.96	2.70	14.33
P5	84.87	2.79	12.85
P6	40.75	2.63	10.82
P7	41.48	2.49	10.24
P8	52.30	2.00	8.50
P9	1.00	0.89	3.29
P10	305.88	5.45	20.45
Pz2	32.70	1.00	3.08
Pz7	6.19	0.38	1.00

## Appendix A

**Table A1:** Partial contamination indices.

Piezometer	Parameter	Background—P9 (Min, Max, Mean)			Background—Pz2 (Min, Max, Mean)			Background—Pz7 (Min, Max, Mean)		
P1	EC	−0.34	1.02	−0.14	0.34	3.53	0.74	1.64	7.96	2.45
	Na	−0.17	1.36	0.31	1.01	4.73	2.19	23.71	69.47	38.2
	Fe	1.8	491	121.95	−0.98	2.25	−0.19	−0.90	16.45	3.36
	Cl	−0.3	1.83	0.14	0.22	3.92	0.99	4.12	19.66	7.34
	SO_4_	−0.78	0.49	−0.55	−0.26	3.89	0.54	−0.05	5.29	0.99
P2	EC	−0.3	1.25	−0.11	0.41	3.06	0.79	1.78	7.04	2.54
	Na	−1.01	0.32	−0.54	0.83	4.73	1.52	21.54	69.47	30.01
	Fe	96.2	435	143.81	−0.36	2.25	0.03	2.45	16.45	4.53
	Cl	−0.47	1.51	0.006	−0.08	3.92	0.85	2.86	19.66	6.78
	SO_4_	−0.67	0.69	−0.3	0.08	3.89	1.23	0.39	5.29	1.87
P3	EC	−0.2	3.81	0.71	0.61	8.66	2.45	2.18	18.12	5.82
	Na	−1.0	3.34	0.6	−1.00	9.55	2.89	−1.00	128.75	46.79
	Fe	6.6	319	119.59	−0.95	1.11	−0.20	−0.73	10.35	3.28
	Cl	−0.14	2.38	0.57	0.5	4.89	1.74	5.29	23.73	10.51
	SO_4_	−0.66	0.69	−0.39	0.11	4.55	0.99	0.11	4.55	0.99
P4	EC	−0.26	1.34	0.23	0.48	3.71	1.47	1.93	8.32	3.89
	Na	−0.12	1.45	0.56	1.13	4.95	2.8	25.22	72.14	45.7
	Fe	185	595	376.8	0.23	2.94	1.5	5.6	20.13	12.4
	Cl	0.02	2.21	0.88	0.77	4.59	2.28	6.43	22.47	12.76
	SO_4_	−0.87	0.25	−0.46	−0.59	3.11	0.79	−0.59	3.11	0.79
P5	EC	−0.21	1.01	0.13	0.58	3.04	1.27	2.13	7	3.48
	Na	−0.33	1.48	0.31	0.62	5.02	2.19	18.87	72.98	38.23
	Fe	151	639	390.58	0	3.23	1.59	4.39	21.7	12.89
	Cl	−0.1	2.28	0.46	0.57	4.71	1.55	5.58	23	9.7
	SO_4_	−0.28	0.65	−0.05	1.35	4.41	2.12	1.35	4.41	2.12
P6	EC	−0.27	0.87	0.19	0.47	2.75	1.4	1.91	6.42	3.75
	Na	−0.25	0.78	0.19	0.82	3.33	1.9	21.38	52.27	34.67
	Fe	36.6	355	184.58	−0.75	1.35	0.23	0.33	11.62	5.58
	Cl	−0.29	1.28	0.38	0.23	2.96	1.4	4.18	15.62	9.07
	SO_4_	−0.51	1.31	0.17	0.61	6.57	2.84	0.61	6.57	2.84
P7	EC	−0.04	0.35	0.16	0.92	1.71	1.34	2.8	4.36	3.63
	Na	−0.28	0.55	0.1	0.74	2.76	1.67	20.37	45.26	31.78
	Fe	42.2	279	188.24	−0.71	0.85	0.25	0.53	8.93	5.71
	Cl	−0.06	0.75	0.33	0.64	2.04	1.31	5.88	11.76	8.72
	SO_4_	−0.51	0.9	0.08	0.61	5.23	2.53	0.61	5.23	2.53
P8	EC	−0.42	0.47	−0.02	0.16	1.95	0.97	1.3	4.84	2.89
	Na	−0.75	0.73	−0.12	−0.38	3.21	1.15	6.58	50.77	25.4
	Fe	30.8	607	239.87	−0.79	3.02	0.59	0.13	20.56	7.54
	Cl	−0.94	0.87	−0.12	−0.90	2.25	0.54	−0.58	12.64	5.46
	SO_4_	−0.91	3.93	−0.17	−0.69	15.16	1.71	−0.69	15.16	1.71
P9	EC	1	1	1	−1	1.54	0.01	−1	4.03	0.99
	Na	1	1	1	−1	3.06	0.22	−1	48.93	13.94
	Fe	1	1	1	−1	−0.97	−1.00	−1	−0.82	−0.99
	Cl	1	1	1	−1	2.05	−0.13	−1	11.79	2.65
	SO_4_	1	1	1	−1	8.87	0.64	−1	8.87	0.64
P10	EC	−0.34	1.21	0.42	0.34	3.44	1.86	1.65	7.79	4.66
	Na	−0.44	0.53	0.02	0.36	2.72	1.47	15.67	44.76	29.34
	Fe	465	2719	1420.42	2.08	16.97	8.39	15.52	95.45	49.4
	Cl	−0.16	0.84	0.28	0.46	2.2	1.22	5.12	12.43	8.33
	SO_4_	−0.43	5.15	1.32	0.88	19.17	6.85	0.88	19.17	6.85
